# As-Doped h-BN Monolayer: A High Sensitivity and Short Recovery Time SF_6_ Decomposition Gas Sensor

**DOI:** 10.3390/s22134797

**Published:** 2022-06-24

**Authors:** Yunfeng Long, Sheng-Yuan Xia, Liang-Yan Guo, Yaxiong Tan, Zhengyong Huang

**Affiliations:** State Key Laboratory of Power Transmission Equipment and System Security and New Technology, School of Electrical Engineering, Chongqing University, Chongqing 400044, China; 15200879239@163.com (Y.L.); xiashengyuan@cqu.edu.cn (S.-Y.X.); guoliangyanself@163.com (L.-Y.G.); huangzhengyong@cqu.edu.cn (Z.H.)

**Keywords:** SF_6_ decomposition gas, gas sensor, As-BN monolayer, DFT

## Abstract

SF_6_ is a common insulating medium of gas-insulated switchgear (GIS). However, it is inevitable that SF_6_ will be decomposed due to partial discharge (PD) in GIS, which will cause hidden dangers to the safe and stable operation of equipment. Based on the DFT method, the two-dimensional nano-composite As-doped h-BN (As-BN) monolayer was proposed. By modeling and calculating, the ability of an As-BN monolayer as a specific sensor for SO_2_F_2_ (compared with an H_2_O adsorption system and CO_2_ adsorption system) was evaluated by parameters such as the binding energy (*E*_b_), adsorption energy (*E*_ads_), transfer charge (Δ*Q*), geometric structure parameters, the total density of states (TDOS), band structure, charge difference density (CDD), electron localization function (ELF), sensitivity (*S*), and recovery time (*τ*). The results showed that an As-BN monolayer showed strong adsorption specificity, high sensitivity, and short recovery time for SO_2_F_2_ gas molecules. Therefore, the As-BN monolayer sensor has great application potential in the detection of SF_6_ decomposition gases.

## 1. Introduction

SF_6_ gas is widely used in gas-insulated switchgear (GIS) due to its high dielectric strength, excellent arc extinguishing ability, and good insulation characteristics [1,2,3]. However, some insulation defects, such as partial discharge (PD), are inevitable during the long-term operation of GIS. The PD is the fault characteristic phenomenon before the complete breakdown or flashover of GIS insulation, and it is also the early manifestation of GIS internal insulation defects, causing great hidden dangers to the safe and stable operation of GIS. Under the continuous action of PD, a variety of low-fluorine sulfides formed by SF_6_ decomposition will react with H_2_O molecules, which will lead to the decomposition of the SF_6_ gas which is difficult to reduce, and generate SO_2_F_2_ and other SF_6_ decomposition gases [4,5,6,7]. This will significantly reduce the insulation performance of SF_6_ gas and accelerate the deterioration of GIS insulation, which may lead to sudden faults in the operation of GIS. Therefore, the monitoring of SF_6_ decomposition gas is of great significance to the defect identification and early warning of GIS.

At present, the traditional methods for detecting SF_6_ decomposition gases produced by PD in GIS include chromatography and spectroscopy [8,9,10,11]. The basic principle of chromatography is to push the mixed gas samples taken from GIS into the chromatographic column, separate SF_6_ decomposition gases by using different gas adsorption or dissolution capacities of each component and identify and calibrate them with special detectors. However, this method is relatively complex, high instrument price has high requirements for operators, and cannot realize online monitoring. Spectroscopy is a quantitative detection of SF_6_ decomposition gases by using the relationship between the absorption degree of different measured gases and the volume fraction of the gas. However, the spectral method has low sensitivity and low detection accuracy in the detection of trace gases, and there is a cross-interference between the absorption peak of SF_6_ and its decomposed components. More importantly, with both spectroscopy and chromatography, it is difficult to achieve online monitoring of SF_6_ decomposition gases. Therefore, it is very important to develop real-time, accurate, convenient, and intelligent SF_6_ decomposition gas monitoring technology.

With the rapid development of nanotechnology, the gas sensor method has made rapid progress. The basic principle of gas sensors based on nanomaterials is that when detecting gas, nanomaterials, such as graphene, boron nitride, and carbon nanotubes, interact with gas, resulting in varying degrees of electrical signal response to calculate the type, concentration, and gas production rate of gas [12,13,14,15,16]. It has the advantages of low preparation cost, simple process, fast detection speed, high sensitivity, and real-time monitoring. At present, most studies on the monitoring of SF_6_ decomposition gases by gas sensor method are mainly common gases, such as SO_2_ and H_2_S, but the exploration of important gas components such as SO_2_F_2_ is still relatively small [17,18,19,20]. Therefore, it is necessary to further develop gas sensors that can cover SO_2_F_2_ and other major SF_6_ decomposition gases. With the development of computer science, the DFT method based on quantum mechanics has been widely used in the field of gas sensing [21,22,23,24,25,26,27,28,29,30,31,32]. According to previous research, the material properties calculated by the DFT method have high consistency with the experimental results, which confirms the feasibility of this method in the field of gas sensing.

In this study, we proposed a novel As-doped h-BN (As-BN) monolayer gas sensor for SF_6_ decomposition gas monitoring. Based on the DFT method, the gas sensing response mechanism of the interaction between As-BN monolayer and SO_2_F_2_ gas molecules was discussed at the micro-level. Considering the fact that SF_6_ gas molecules are more likely to produce SO_2_F_2_ gas in the presence of gases such as H_2_O molecules in the air, the gas sensing response parameters with As-BN monolayer and H_2_O and CO_2_ gas molecules are also explored. This study provides a theoretical basis for the preparation of an As-BN monolayer gas sensor for SF_6_ decomposition gas detection and provides a convenient way for the development of other sensors.

## 2. Computational Details

Based on the DFT method, the construction and calculation of the model in this study are all in DMol3 and CASTEP codes of the Materials Studio software [33,34,35,36]. In order to avoid the interference of adjacent units, a 15 Å vacuum layer was constructed. The Perdew-Burke-Ernzehof (PBE) of the generalized gradient approximation (GGA) is selected to better deal with the inter-electron exchange-correlation functional. DFT semi-core pseudo-potential (DSPP) is used to simplify the electronic interaction between atoms. At the same time, double numerical plus polarization (DNP) is also added as the basic function of the linear combination method of atomic orbitals. Considering the Van Der Waals forces in the process of impurity doping and gas adsorption and the interaction between long distances, the DFT-D2 method is used to analyze all models, which can effectively improve the calculation accuracy of the system. The k point, energy convergence accuracy, maximum force, and maximum displacement are set to 6 × 6 × 1, 1 × 10^−5^ Ha, 2 × 10^−3^ Ha/Å, 5 × 10^−3^ Å.

In this study, the calculation formula of binding energy is as follows:

*E*_b_ = *E*_doped-BN_ + *μ*_B/N_ − *E*_BN_ − *μ*_As_ (1)

where *E*_doped-BN_ and *E*_BN_ represent the energy of the doped-BN monolayer and pristine h-BN monolayer, respectively; the μ represents the chemical potential of the counterpart elements. The calculated formation energies of As-BN monolayers doped with As atoms at positions 1 and 2 are 6.501 eV and 6.931 eV, respectively.

In this study, the adsorption energy formula is as follows:

*E*_ads_ = *E*_Gas/As-BN_ − *E*_Gas_ − *E*_As-BN_ (2)

where *E*_Gas/As-BN_, *E*_Gas_, and *E*_As-BN_ represent the energy of adsorption systems, gas molecule, and As-BN monolayer, respectively.

In this study, the calculation formula of transfer charge is as follows:

Δ*Q* = *Q*_1_ − *Q*_2_ (3)

where *Q*_1_ and *Q*_2_ represent the total charge of the gas molecule after and before adsorption, respectively.

In this study, the calculation formula of sensitivity is:

*S* = (1/*σ*_As-BN/gas_ − 1/*σ*_As-BN_)/(1/*σ*_As-BN_) (4)

where the *σ*_As-BN/gas_ and *σ*_As-BN,_ respectively, represent the conductivity of adsorption systems and As-BN monolayer.

In this study, the calculation formula of recovery time is:

*τ* = *v*_0_^−1^ exp(−*E*_ads_/kT) (5)

where *v*_0_, k, and T represent the attempt frequency, Boltzmann constant, and thermodynamic temperature, respectively.

## 3. Results and Discussion

**Figure 1:**
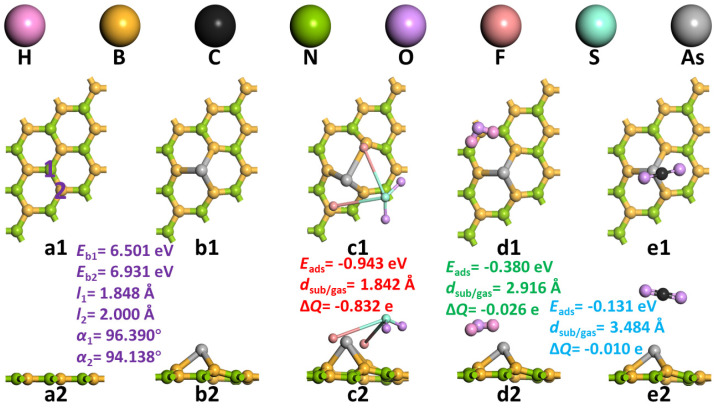
The geometric structures of (**a1**,**a2**) h−BN monolayer, (**b1**,**b2**) As−BN monolayer, (**c1**,**c2**) SO_2_F_2_ adsorption system, (**d1**,**d2**) H_2_O adsorption system, (**e1**,**e2**) CO_2_ adsorption system.

Constructing appropriate h-BN monolayers is the basis for our subsequent research. As shown in Figure 1(a1,a2), we first constructed h-BN monolayers containing nine N atoms and nine B atoms in a 3 × 3 × 1 supercell. The B-N bond length is 1.453 Å, and the B-N-B or N-B-N bond angle is about 120°. At the same time, other parameters are also similar to previous studies. Then, we doped the h-BN monolayer by substituting an As atom for an N atom (position 1) or a B atom (position 2). At the same time, we calculated the geometric structure parameters and binding energy (*E*_b_) of the two after doping. Both are positive, indicating that the formation reactions of the two are endothermic reactions. The formation of the two needs to provide heat or other forms of energy in the outside world. The smaller formation energy of position 1 indicates that the doping method of one As atom instead of one N atom is more reliable and stable, while the doping method of position 2 is difficult to complete in the experiment. This may be related to the fact that an As atom has the same valence electron as an N atom. This makes an As atom replaces an N atom without an unpaired electron, and an As atom replacing a B atom will produce defects. From the perspective of geometric configuration, the B-N bond length of the b-BN monolayer (1.453 Å) is closer to the As-B bond length after doping in position 1 (*l*_1_ = 1.848 Å) than that after doping in position 2 (*l*_2_ = 2.000 Å). This indicates that the binding of an As atom at doping site 1 is closer than that at doping site 2, the change of bond length before doping is smaller, and a more stable structure can be formed. At the same time, the B-As-B bond angle (*α*_1_ = 96.390°) formed by doping site 1 is closer to the B-N-B/N-B-N bond angle (120°) before doping than the N-As-N bond angle (*α*_2_ = 94.138°) formed by doping site 2. The large change of bond angle will cause excessive distortion of the material, which will not be conducive to the stability of the material and the application of the function. Therefore, the adsorption of the three gas molecules (SO_2_F_2_, H_2_O, and CO_2_) explored in this study was based on an As atom to replace an N atom of the As-BN monolayer, as shown in Figure 1(b1,b2).

Three gases (SO_2_F_2_, H_2_O, and CO_2_) were close to the As-BN monolayer from different orientations and positions to obtain the optimal adsorption configuration, as shown in Figure 1(c1–e2). In order to quantify the adsorption of As-BN monolayer on various gases, the adsorption energies (*E*_ads_), the nearest distance of gas molecules to the substrate (*d*_sub/gas_), and the transfer charge (Δ*Q*) were calculated.

From the perspective of adsorption energy, the adsorption energy of the three adsorption systems is negative, and the adsorption effect of the As-BN monolayer on SO_2_F_2_ gas molecules is significantly stronger than that of the other two gas molecules. This indicates that the three adsorption reactions are exothermic and spontaneous, and the adsorption effect of the As-BN monolayer on SO_2_F_2_ gas molecules is particularly strong. In general, we consider that an adsorption energy of less than −0.6 eV is chemical adsorption. This means that the adsorption time and detection time of an As-BN monolayer for SO_2_F_2_ gas molecules will reach a good balance. The other two gases may be desorbed from the As-BN monolayer without an electrical signal display. This proves the specificity of the As-BN monolayer for SO_2_F_2_ adsorption. From a geometrical point of view, the As-BN monolayer adsorption of SO_2_F_2_ gas molecules is more obvious than the change in H_2_O gas molecules and CO_2_ gas molecules, as shown in Figure 1(c1–e2). Before and after adsorption, the bond lengths of SO_2_F_2_, H_2_O, and H_2_O molecules changed from 1.613 Å of S-F bond length to 3.724 Å, 0.97 Å of H-O bond length to 0.972Å, and 1.176 Å of C=O bond length to 1.175 Å, respectively. It can be found that the change of bond angle is also similar, and the geometric configuration change of the SO_2_F_2_ gas molecule is more obvious than that of the other two gases. At the same time, the shortest distance between SO_2_F_2_ gas molecules and the substrate is also smaller. This indicates that the adsorption of SO_2_F_2_ gas molecules on the As-BN monolayer is closer and stronger, which may lead to a more obvious electrical signal response of the As-BN monolayer. It is worth mentioning that the shortest distance between the three gas molecules and the substrate is the shortest distance between an atom of the gas molecule and an As atom. This indicates that doping an As atom to replace an N atom will significantly improve the gas-sensing adsorption capacity of the substrate. From the perspective of transfer charge, the transfer charge of the SO_2_F_2_ gas adsorption system is 40 and 80 times that of the other two adsorption systems. This will make the electrical signal response of the As-BN monolayer before and after the adsorption of different gases show obvious differences. Therefore, from the above point of view, compared with CO_2_ gas molecules and H_2_O gas molecules, the As-BN monolayer can achieve good specificity detection for SO_2_F_2_ gas molecules.

**Figure 2:**
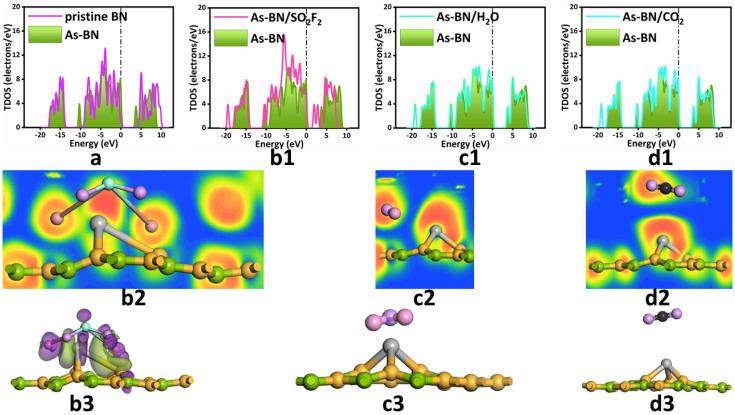
(**a**) The TDOS of h−BN monolayer and As−BN monolayer. The TDOS, ELF, and CDD of (**b1**–**b3**) SO_2_F_2_ adsorption system, (**c1**–**c3**) H_2_O adsorption system, (**d1**–**d3**) CO_2_ adsorption system. The Fermi level is set at zero.

In order to further explore the electronic behavior of each model, we calculated their total density of states (TDOS), electron localization-focusing function (ELF), and charge difference density (CDD), as shown in Figure 2. Figure 2a describes the TDOS of the substrate before and after doping. It can be seen that the As atom doped As-BN monolayer TDOS overall moves to the lower left energy direction, but the change of the peak size is relatively small, indicating that the doped As atoms have no significant effect on the crystal structure of the substrate. Continuous TDOS means that the As-BN monolayer has good conductivity. The TDOS of the As-BN monolayer has an obvious peak at the Fermi level, indicating that the energy gap of the As-BN monolayer doped with an As atom instead of an N atom is smaller than that of the h-BN monolayer, which makes electrons more prone to transition. Therefore, the doping of As atoms is beneficial to improving the conductivity of the h-BN monolayer. At the same time, there are different degrees of hybridization and overlap in some energy levels, which shows that As atoms can form a stable doping structure with an h-BN monolayer.

Figure 2(b1,c1,d1) shows the TDOS of three gas adsorption systems. Compared with the other two adsorption systems, the change of TDOS in the SO_2_F_2_ gas adsorption system before and after adsorption is more obvious, as shown in Figure 2(b1). Near the Fermi level, TDOS changes significantly, which enhances the electron co-ownership movement around SO_2_F_2_ gas molecules and enhances the electron transition ability between the valence band and conduction band. The electrical conductivity of the system was significantly improved. At the same time, TDOS increases significantly at the far Fermi level, which may also contribute to conductivity. However, the TDOS of the other two adsorption systems did not change significantly before and after adsorption, especially near the Fermi level. This indicates that it is difficult to monitor the adsorption of H_2_O or CO_2_ molecules on the As-BN monolayer surface. Even if there are new peaks or some changes in TDOS far away from the Fermi level, the contribution to conductivity is negligible. This is also consistent with the small adsorption energy of these two gas molecules in the adsorption process.

The above analysis was also confirmed in the ELF and CDD of the three adsorption systems, as shown in Figure 2(b2,b3). The fusion of orange and green regions indicates that the F atom and As atom of the SO_2_F_2_ gas molecule have strong mutual attraction, and the electron density increases significantly, but the two are obviously not completely fused. Combined with the adsorption effect in the SO_2_F_2_ adsorption system previously analyzed, the adsorption between SO_2_F_2_ gas molecules and As-BN monolayer is more likely to be between physical adsorption and chemical adsorption. Figure 2(c2,d2) showed that the ELF of the H_2_O adsorption system and CO_2_ adsorption system also tend to converge, but this trend is weaker than that of the SO_2_F_2_ adsorption system. Therefore, combined with the previous adsorption energy, transfer charge, and geometric configuration parameters, the adsorption between the H_2_O gas molecule and the CO_2_ gas molecule and the As-BN monolayer were determined as physical adsorption. In the CDD of the SO_2_F_2_ gas adsorption system, there are dense electron concentration areas and electron dissipation areas around gas atoms and As atoms, as shown in Figure 2(b3). This indicates that the adsorption reaction of the two is accompanied by intense charge transfer. This is also very consistent with the previously calculated charge transfer (−0.832 e). At the same time, the atoms of SO_2_F_2_ gas molecules are more surrounded by electron concentration areas, which is also consistent with the negative charge transfer calculated previously. The SO_2_F_2_ gas molecule is an electron acceptor, and the As-BN monolayer is an electron donor. However, the electron-dissipation region and electron-aggregation region of the other two adsorption systems are not obvious at the same isosurface value as the SO_2_F_2_ adsorption system. This proves that the charge transfer between H_2_O and CO_2_ molecules interacting with the As-BN monolayer is very small. This is consistent with the previous calculation of charge transfer; that is, the charge transfer of the H_2_O adsorption system and the CO_2_ adsorption system is much smaller than that of SO_2_F_2_. Therefore, in the three adsorption systems, the adsorption and electronic behavior of the As-BN monolayer on SO_2_F_2_ gas molecules are particularly strong.

**Figure 3:**
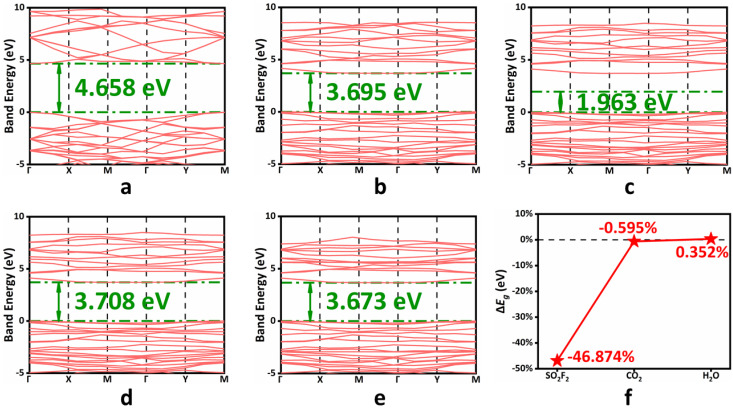
The band energy of (**a**) h−BN monolayer, (**b**) As−BN monolayer, (**c**) SO_2_F_2_ adsorption system, (**d**) H_2_O adsorption system, (**e**) CO_2_ adsorption system. (**f**) The Δ*E*_g_ of three adsorption systems.

For resistive gas sensors, it is necessary to detect SO_2_F_2_ gas molecules when the conductivity changes properly. Band energy has a significant correlation with the conductivity of the system. When the band gap increases, the conductivity of the system decreases; when the band gap decreases, the conductivity of the system increases. The larger the band gap, the harder it is for electrons to enter the conduction band. Reflected in the macro are the current or voltage changes. It can be seen from Figure 3a,b that the band gap of the As-BN monolayer doped with As atoms decreased significantly from 4.658 eV to 3.695 eV. The decrease in the band gap means that the electron transition will be easier, and the change in the conductivity of the material may be more obvious. As shown in Figure 3b–f, after the adsorption of the SO_2_F_2_ gas molecules, the energy gap value of the system changes significantly and is significantly different from that of the other two adsorption systems, and the band gap energy decreases by nearly 50%. The absolute values of the band gap energy changes of the other two adsorption systems are less than 0.6%, which is very difficult in the actual detection. The specificity of the As-BN monolayer for SO_2_F_2_ gas molecular detection was also proved. At the same time, we consider that SF_6_ is more likely to decompose into characteristic gases such as SO_2_F_2_ under the condition of micro-water. Therefore, the distinguishing detection of H_2_O and SO_2_F_2_ gas molecules are particularly important in SF_6_ decomposition gas detection. Therefore, the As-BN monolayer is very suitable for the detection of SF_6_ decomposition gases.

**Figure 4:**
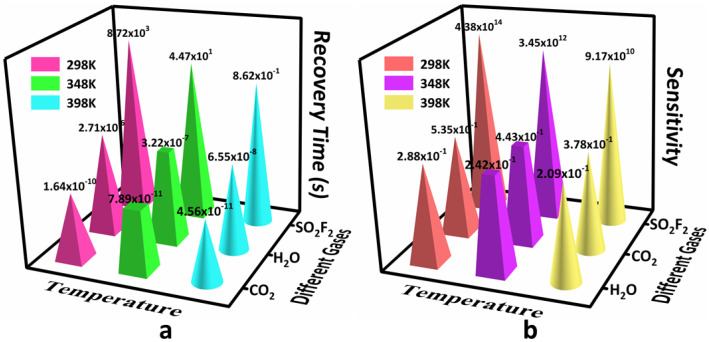
The (**a**) recovery time and (**b**) sensitivity of three adsorption systems.

In order to further explore the practical potential of the As-BN monolayer gas sensor, the sensitivity (*S*) and recovery time (*τ*) of three adsorption systems were calculated. Sensitivity is an important performance index of gas sensors, which is closely related to the change of band energy gap of the system. Recovery time is an important parameter to describe the desorption rate of the gas sensor to the target gas, which is closely related to the adsorption energy when the gas sensing material interacts with the measured gas. Figure 4a shows the recovery time of three adsorption systems. The results showed that the recovery time was from 44.7 s to 0.862 s at the temperature of 358 K to 398 K. This was because the adsorption and desorption of the As-BN monolayer and SO_2_F_2_ gas were easier with the increase in temperature due to the addition of external energy. At the same time, the recovery time of the other two adsorption systems is shorter. This makes it impossible for the As-BN monolayer to effectively adsorb and respond to H_2_O or CO_2_ gas molecules in practical applications. This is very useful for the specific detection of SO_2_F_2_ gas. As shown in Figure 4b, the SO_2_F_2_ gas adsorption system shows high sensitivity at various temperatures. The sensitivity is 4.38 × 10^14^ at room temperature (298 K). At 398 K, the sensitivity of the As-BN monolayer gas sensor can also reach 9.17 × 10^10^. This means that in the early stage of PD, the As-BN monolayer gas sensor can detect SO_2_F_2_ gas in time, which provides the possibility for early warning. In conclusion, the As-BN monolayer has great potential for the detection of SF_6_ decomposition gases.

## 4. Conclusions

In this study, the h-BN monolayer, As-BN monolayer, and three gas molecular models (SO_2_F_2_, H_2_O, and CO_2_) were established, and the optimal adsorption structures of the three gases were obtained through calculation and analysis. On this basis, by calculating the adsorption energy, geometric structure parameters, transfer charge, adsorption energy, TDOS, ELF, CDD, band structure, recovery time, and sensitivity of each system, the possibility of an As-BN monolayer as a sensitive layer to detect the important characteristic gas SO_2_F_2_ in SF_6_ decomposition gases was analyzed. The calculation results show that in the three adsorption systems, the parameters of the SO_2_F_2_ adsorption system are more appropriate and significantly different from those of the other two adsorption systems, which ensures the specificity of the As-BN monolayer for the detection of SO_2_F_2_ gas molecules. At the same time, at multiple temperatures. The detection of SO_2_F_2_ gas molecules by the As-BN monolayer can ensure high sensitivity and short recovery time. Therefore, this study not only provides a theoretical basis for the preparation of As-BN monolayer gas sensors for SF_6_ decomposition gas monitoring but also provides a convenient way for the development of other sensors.

## Data Availability

Not applicable.
